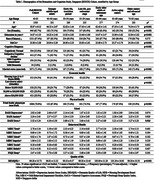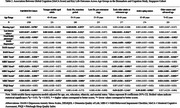# Impact of Global Cognition on Mental Health, Physical Measures and Economic Health Differs Across Age Bands: Findings from the Southeast Asian BIOCIS Study

**DOI:** 10.1002/alz70861_108728

**Published:** 2025-12-23

**Authors:** Yi Jin Leow, Justin Jit Hong Ong, Jia Dong James Wang, Nagaendran Kandiah

**Affiliations:** ^1^ Dementia Research Centre (Singapore), Lee Kong Chian School of Medicine, Nanyang Technological University, Singapore Singapore; ^2^ Lee Kong Chian School of Medicine, Nanyang Technological University, Singapore Singapore; ^3^ Lee Kong Chian School of Medicine, Singapore, Singapore Singapore; ^4^ Lee Kong Chian School of Medicine, Nanyang Technological University, Singapore, Singapore Singapore; ^5^ Neuroscience and Mental Health Programme, Lee Kong Chian School of Medicine, Nanyang Technological University, Singapore Singapore; ^6^ National Healthcare Group, Singapore Singapore; ^7^ Duke‐NUS Medical School, National University of Singapore, Singapore Singapore

## Abstract

**Background:**

Cognition shapes mood, behavior, health, and wealth. While cognitive decline in older age is frequently linked with mood and functional impairment, the influence of global cognition on broader life outcomes from across the lifespan remains underexplored. This study examines how global cognition relates to mental health, behavioral patterns, economic stability, physical frailty, and quality of life(QOL) across distinct adult life stages.

**Methods:**

We analyzed baseline data from 1,500 community‐dwelling adults aged 40–85 in Singapore’s BIOCIS study, spanning cognitive statuses from normal to mild dementia. Measures included global cognition (MoCA), mood (DASS), behavior (MBI‐C, PSQI), quality of life (DEMQOL), economic health (housing type, salary), and frailty (Fried Frailty). Multivariable regressions examined associations between cognition and outcomes, stratified by clinically meaningful age bands and adjusted for demographic covariates.

**Results:**

Participants (mean age 59.87±9.13years) were predominantly female (62.7%) and Chinese (87.4%). With age, education and MoCA scores declined, while cognitive impairment prevalence increased (*p* <0.001). Younger adults had better economic stability, higher stress, and lower frailty, whereas older adults had greater behavioral symptoms and frailty (*p* =0.016 to *p* <0.005). QOL peaked at 60–64 years before declining (*p* <0.001). Across the entire cohort, higher global cognition correlated significantly with better housing conditions, higher salaries, lower frailty, and enhanced quality of life (allp<.05). Conversely, improved cognition inversely correlated with MBI‐C‐Total and domains of poor‐mood, impulse‐dyscontrol, social‐appropriateness, and abnormal‐beliefs (all *p*<.005). Notably, these associations varied by life stage. In younger adults (40–49,50–54), higher cognition was linked to higher salary (*p* <0.05), with the 50–54 group also showing better housing (*p* <0.05), fewer abnormal beliefs (*p* <0.05), and less social‐inappropriateness (*p* <0.005). In mid‐to‐older adults (60–64), higher MoCA was associated with higher salary (*p* <0.005), better QOL (*p* <0.05), and lower MBI‐C‐Total and subdomains of mood, impulse‐dyscontrol, social‐inappropriateness and abnormal‐beliefs (*p* <0.05top<0.005). In those aged 65–69, cognition correlated with better QOL (*p* <0.05), and in the 70–85 group, with lower frailty (*p* <0.05) and consistently lower MBI‐C‐Total and all MBI‐C‐subdomains (all *p*<0.005).

**Conclusion:**

Cognitive health impacts mental, behavioral, physical, and economic well‐being, with variations across life stages. Economic stability is closely tied to cognition in midlife, while mood, behavioral disturbances, and frailty dominate in later years. Associations emerge by midlife (60–64), highlighting a window for preventive strategies. These findings underscore a public health imperative—where promoting brain health in midlife may mitigate risks of Alzheimer's disease and improve life outcomes across aging trajectories.